# Establishing a Mouse Model of *NL3*^R617W^-Associated Autism Spectrum Disorder for a Functional Study

**DOI:** 10.62641/aep.v53i2.1780

**Published:** 2025-03-05

**Authors:** Wei Gao, Qiao Cai, Xiaoming Ying, Bei Zhao

**Affiliations:** ^1^Children’s Health Department, Hongkou District Maternal and Child Health Institution, 200000 Shanghai, China; ^2^Department of Pediatric, The First People’s Hospital of Taizhou, 318020 Taizhou, Zhejiang, China

**Keywords:** autism, neuroligin-3, hippocampal, 293T cell, synaptic protein

## Abstract

**Background:**

Autism spectrum disorder (ASD) is a neurodevelopmental disorder characterized by deficits in social communication and limited behavior. Despite the association of numerous synaptic gene mutations with ASD, the presence of behavioral abnormalities in mice expressing autism-associated R617W mutation in synaptic adhesion protein neuroligin-3 (NL3) has not been established. This work focuses on establishing a mouse model of ASD caused by *NL3* R617W missense mutation (*NL3*^R617W^) and characterizing and profiling the molecular as well as behavioral features of the animal model.

**Methods:**

The expression and distribution of *NL3*^R617W^ mutant protein in the 293T cell membrane and intracellular NL3 was detected by using immunofluorescence approach. Meanwhile, synaptic markers (Synapsin I, vesicular glutamate transporter (VGluT) I and vesicular γ-aminobutyric acid transporter (VGAT)) and synapse number were detected with a confocal fluorescence microscope. Thereafter, the effect on *NL3*^R617W^ was verified. The expression of synaptic proteins, postsynaptic density protein-95 (PSD95) and Src homology domain and multiple ankyrin repeat domains protein 3 (SHANK3), was verified by Western blot. The interaction between NL3 and neurexin 1 (NRXN1) was studied by means of co-immunoprecipitation. The behavior of autistic mice induced by *NL3*^R617W^ mutation was examined using the Morris water maze and the Y maze. *NL3*^R617W^ mutant mice were assessed in the open field, and three-chamber test was conducted to assess and observe the presence of hyperactivity, repetitive behavior, friendliness, and social novelty.

**Results:**

The results indicated that the *NL3* mutation could influence the interaction between NL3 and NRXN1, and inhibit the expression of VGluT I. Nevertheless, *NL3* mutation would not influence the expression of NL3 on cell membrane, the intracellular distribution of NL3, or the endoplasmic reticulum retention. The outcomes of animal studies demonstrated that the ASD mice with *NL3*^R617W^ exhibited a significant decrease in the capacity for spatial memory and exploration, as well as the expression levels of the postsynaptic scaffolding proteins, PSD95 and SHANK3 (*p* < 0.05). The number of excitatory synapses in hippocampal cornu ammonis (CA)1 and CA3 and the sensory cortex was also significantly reduced (*p* < 0.01). Compared to the control mice, the *NL3*^R617W^ mutant mice were less active in the open field (*p* < 0.001), a finding consistent with the three-chamber test result showing reduced degree of activity. Furthermore, compared to the control mice, the *NL3*^R617W^ mutant animals spent less time with stranger mice (*p* < 0.05).

**Conclusions:**

*NL3*^R617W^ mutation may inhibit the expression of postsynaptic scaffolding proteins by influencing the interaction with *NRXN1*, thus inhibiting synapse formation and reducing the number of excitatory synapses.

## Introduction

Autism is categorized under the umbrella of autism spectrum disorder (ASD), which manifests in the early stages of development, typically before the age of three, and is marked by unusual repetitive and/or restricting behaviors or interests and a significant impairment in social communication [1, 2]. Following the identification of nearly 800 susceptibility, clinically relevant, numerous etiological studies, including animal experiments [3], on these genes, no definite pathogenic mechanisms, biomarkers [4], or particular mode of transmission for the development of autism has been firmly established [5]. Passing a water maze test is challenging for animals because they need to develop and employ several non-spatial skills to succeed in the test, in addition to navigating the hidden platform using their spatial skills [6]. Accordingly, potential neurological issues can be pinpointed by examining how animals pay attention and learn in behavioral tests.

ASD is primarily a hereditary disorder, associated with multiple genes, and several mouse models of ASD have been developed [7]. The majority of the genes linked to ASD are believed to encode synaptic proteins, including neuroligin, neurexin, and synaptic cell adhesion molecules, as well as Src homology domain and multiple ankyrin repeat domains protein 3 (SHANK/ProSAP), a scaffold protein found in the postsynaptic density (PSD) [8]. Four isoforms of postsynaptic cell-adhesion molecules known as neuroligins interact with presynaptic neurexin to play a role in the establishment and maintenance of synapses. Mutations in the neuroligin-3 (*NL3*) and neuroligin-4 genes are among the several gene abnormalities that are associated with ASD [7]. NL3 is present at excitatory and inhibitory synapses [9]. The currently known *NL3* mutations associated with autism include R451C [10], G426S [11], V321A [12], P514S, and R597W [13]. Well-validated in *in vivo* and *in vitro* models of autism, these mutations have been found to affect the learning and cognition in mice, reduce synaptic excitability, and produce oxidative stress. Autism caused by mutations in the neuroligin-3 (*NLGN3*) gene may be accompanied by neuronal oxidative stress [14]. Nevertheless, no prior research has been carried out on the impact of the *NL3*^R617W^ mutation in *in vitro* and *in vivo* models of autism.

Neuroligins (NLs) are postsynaptic cell-adhesion molecules that play a role in the development, structure, and remodeling of synapses [15, 16]. A recent study has outlined the novel function of NL3 as a secreted protein [17]. The ɑ-latrotoxin receptors present in the venom of black widow spiders are the source of the neurexin (NRXN) family of single-pass transmembrane proteins [18]. Membrane-associated mucin domain-containing glycosylphosphatidylinositol anchor proteins (MDGAs) block the neuroligin (NLGN2)-promoted γ-aminobutyric-acid-ergic (GABAergic) synapse formation, likely by disrupting the NLGN2–NRXN connection [19, 20]. However, how the *NL3*^R617W^ mutation interferes with the NL3–NRXN interaction remains elusive.

NLs exhibit a robust synaptogenic activity when overexpressed in cultured neurons; however, even in NL1, 2, and 3 triple-knockout animals, minimal influence on the development of synapses in the forebrain has been observed [21]. Calsyntenin-3 binds to ɑ-NRXNs to produce a trans-synaptic complex that initiates excitatory and inhibitory presynapse differentiation [22]. Dynein depletion or inhibition of the interaction with postsynaptic density protein-95 (PSD95) has been demonstrated to reduce NLGN internalization, resulting in increases in the spine head size and length of PSD in synapses [23]. Furthermore, ASD has been linked to mutations in neurexin-1 (which binds to NLs extracellularly) [24], and SHANK3 (which binds to NLs intracellularly via PSD95) has also been associated with ASD [25]. However, whether the R617W point mutation in NL3 influences synaptic elimination and synaptic protein decrease *in vivo* or *in vitro* remains unknown.

In this work, we constructed a model of autism associated with the *NL3*^R617W^ mutation. In addition, NL3 distribution and expression in the cell membrane and within the cell, as well as changes in synaptic excitability and synaptic proteins SHANK3 and PSD95, were evaluated.

## Methods

### Cell Culture

Human renal epithelial cell line 293T (BFN60810479) from Shanghai Chinese Academy of Sciences was cultured in Dulbecco’s modified Eagle medium (DMEM, high-glucose; C11995500BT, Gibco, Life Technologies, Rockville, MD, USA), supplemented with 10% fetal bovine serum (FBS; 10099-141, Gibco, Life Technologies, Rockville, MD, USA) and 1× streptomycin (15140122, Gibco, Life Technologies, Rockville, MD, USA).

Three mice used in this study were purchased from Zhejiang Weitong Lihua Co., Ltd. (Jiaxing, China, 20220824Abzz0600007602). The hippocampus was isolated from C57Bl/6 male mice of 3–4 days of age and placed in cold HEPES-buffered solution (HBS; composition: 130 mM NaCl, 5.4 mM KCl, 1.8 mM CaCl_2_, 1 mM MgCl_2_, 10 mM HEPES, and 25 mM D-glucose; [pH 7.4]) [26]. The tissues were chopped and treated with protease solution containing 1 mg/mL of HBS at room temperature for 30 minutes. They were then washed in HBS, repeatedly ground, and then centrifuged (1000 rmp, 3 min). The resulting cells were cultured in Neurobasal^TM^ medium (21103049, Gibco, Life Technologies, Rockville, MD, USA) supplemented with 2% B27 (17504044B27, Gibco, Life Technologies, Rockville, MD, USA), 1% streptomycin and 1% L-glutamine (25030081, Gibco, Life Technologies, Rockville, MD, USA). This tissue culture process mainly produces astrocytes. All the cells were incubated at 37 °C with 5% CO_2_ and saturated humidity. Mycoplasma was not detected in the cell lines employed in this investigation. The findings indicated no mycoplasma contamination. The short tandem repeat (STR) analysis was used to authenticate and verify the identity of every cell line used. The mice were anesthetized with 1% pentobarbital sodium at a dose of 40 mg/kg and euthanized with neck dislocation [27].

### Plasmids and Cell Transfection

NL3-HA-wild type plasmid (WT; 5^′^-TTCTCCGAACGTGTCACGT-3^′^), NL3-HA-R617W interference plasmid (5^′^-GGCGAGGACTTAGCGGATAAT-3^′^), and neurexin 1 (NRXN1) expression plasmid were purchased from Hangzhou Guannan Biology (Hangzhou, China). Transfection was performed when the 293T cells reached about 80% confluence; the cells were cultured in fresh and complete DMEM medium about 1 h before transfection. The 293T cells were transfected with NL3-HA-WT and NL3-HA-R617W using Lipofectamine™ 3000 (L3000150, Thermo Fisher, Waltham, MA, USA) and the cells were divided into *NL3*^𝑊𝑇^ group and *NL3*^R617W^ group. After a transfection process for 6 h, the medium was replaced with fresh DMEM complete culture medium, and samples were collected for laser copolymerization experiment or immunoprecipitation for the purpose of protein detection and analysis until 24 h.

NRXN1 expression plasmid: Cctcctaatgaccgacccagtacacgagcagacagactggccataggttttagcactgttcagaaagaagccgtattggtgcgagtggacagttcttcaggcttgggtgactacctagaactgcatatacaccagggaaaaattggagttaagtttaatgttgggacagatgacatcgccattgaagaatccaatgcaatcattaatgatgggaaataccatgtagttcgtttcacgaggagtggtggcaatgccacgttgcaggtggacagctggccagtgatcgagcgctaccctgcagggcgtcagctcacaatcttcaatagccaagcaaccataataattggcgggaaagagcagggccagcccttccagggccagctctctgggctgtactacaatggcttgaaagttctgaatatggcagccgaaaacgatgccaacatcgccatagtgggaaatgtgagactggttggtgaagtgccttcctctatgacaactgagtcaaca

### Co-Immunoprecipitation

The cell samples were rinsed twice with 1× phosphate buffer saline (PBS) (E607008-0500, Sangon Biotech, Shanghai, China) and centrifuged at 1000 rpm for 5 min. Thereafter, 500 µL immunol precipitation (IP) lysis buffer, containing 5 µL of protease inhibitor, was added. The samples were then resuspended, placed on ice for 5 min, and centrifuged at 14,000 rpm at 4 ℃ for 10 min. Subsequently, protein concentration of the samples was measured. The samples were diluted to 1 mg/mL; 50 µL was taken out of each diluted sample as the input, and the rest was used for the IP experiment. Anti-FLAG M2 magnetic beads (M8823, Sigma, St. Louis, MO, USA) and control magnetic beads (P2171, Beyotime Biotechnology, Shanghai, China) were combined with cell lysate and incubated at 4 ℃ overnight. Magnetic rack adsorption was applied to discard the supernatant. Afterward, 1 mL of IP Buffer-II (88804, Thermo Fisher Scientific, Waltham, MA, USA) resuspension magnetic beads were added to absorb the residual liquid. Subsequently, sodium dodecyl sulfate-polyacrylamide gel electrophoresis (SDS-PAGE), membrane transfer, protein detection using primary and secondary antibodies, and Western blotting were performed. The primary antibodies used included anti-FLAG Tag antibody (1:1000, 8146, CST, Boston, MA, USA) and anti-HA antibody (1:1000, 2367, CST, Boston, MA, USA). The secondary antibodies used included goat anti-mouse immunoglobulin G (IgG) (H+L) secondary antibody (1:5000, 32430, Thermo Fisher Scientific, Waltham, MA, USA).

### Western Blot Assay

Approximately 0.1 mg of brain tissue was lysed in radioimmunoprecipitation assay (RIPA) buffer (P0013J-100, Beyotime, Shanghai, China). The protein concentrations were determined by using the bicinchoninic acid assay (BCA) assay (P0012, Beyotime, Shanghai, China). Equal amounts of protein (20 µg) were separated by means of SDS-PAGE (161-0302, Bio-Rad Corporation, Hercules, CA, USA), and the separated proteins were transferred to polyvinylidene fluoride (PVDF) membrane (IPVH00010, Millipore Corporation, Billerica, MA, USA). After blocking the membrane, incubation with primary antibody and then with an appropriate secondary antibody was initiated. The primary antibodies included anti-SHANK3 (1:1000, 64555S, CST, Boston, MA, USA), anti-PSD95 (1:1000, 3409S, CST, Boston, MA, USA), and anti-GAPDH (glyceraldehyde-3-phosphate dehydrogenase) (1:1000, AF0006, Beyotime, Shanghai, China). The secondary antibodies used included goat anti-rabbit IgG (1:5000, GAR0072, LiankeBio, Hangzhou, China) and goat anti-mouse IgG (1:5000, GAM007, LiankeBio, Hangzhou, China). The protein bands were then visualized with enhanced chemiluminescence (ECL) kits (BL520b, Biosharp Life Scinece, Hefei, China) and analyzed using Image J software (v1.34, National Institutes of Health, Bethesda, MD, USA).

### Immunofluorescence Detection

The expression of NL3 in cell membrane was examined. After culture, the cells were fixed and sealed using the Alexandre’s method [28]. Detection of NL3 expression was conducted by detecting HA-TAG (1:1000, 3724S, CST, Boston, MA, USA) on the NL3 protein.

In addition, intracellular distribution of NL3 was also examined. After culture, the cells were labeled with endoplasmic reticulum tracker Green (C1042S, Beyotime, Shanghai, China) and Golgi-tracker Red (C1043, Beyotime, Shanghai, China). Thereafter, the cells were fixed, and the NL3 protein was labeled with hemagglutinin (HA). The corresponding cells were imaged by means of laser confocal microscopy (LSM880, Zeiss, Oberkochen, Baden-Württemberg, Germany).

To detect synaptic changes, neurons were newly extracted and then laid in a transwell sublaminate chamber. The cell density of the laminate was about 65%, and NL3-HA-R617W or NL3-HA-wild plasmid was transfected on the second day. During the neuron extraction, 293T cells in the logarithmic stage of cell growth were obtained to coat 24-well plates with a cell density of about 30%. The cells were transfected with corresponding plasmids in different groups on the following day. After transfection for 24 h, the plates were digested and placed into the upper chamber of the Transwell plates for co-culture with neurons. The expression of Synapsin I (1:1000, ab254349, Abcam, Cambridge, MA, USA), vesicular glutamate transporter (VGluT) I (1:1000, 135307, SYSY, Gottingen, Niedersachsen, Germany), and vesicular GABA transporter (vesicular γ-aminobutyric acid transporter (VGAT), 1:1000, 131004, SYSY, Gottingen, Niedersachsen, Germany) were detected after 5 days of co-culture. The corresponding cell images were captured by laser confocal imaging. Donkey anti-goat IgG secondary antibody (Alexa Fluor™ 555) (A-21432, Thermo Fisher Scientific, Waltham, MA, USA), goat anti-rabbit IgG secondary antibody (Alexa Fluor™ 488) (A-11008, Thermo Fisher Scientific, Waltham, MA, USA), and goat anti-guinea pig IgG secondary antibody (Alexa Fluor™ 647) (A-21450, Thermo Fisher Scientific, Waltham, MA, USA).

For the determination of VGluT I and VGAT in mouse brain cornu ammonis (CA)1, CA3 and sensory cortex, the mouse brain was first fixed with 4% paraformaldehyde (P0099, Beyotime, Shanghai, China). After dehydration, transparency and paraffin embedding, the tissue was cut to a thickness of 4 µm. VGluT I and VGAT were added to the slices and incubated overnight at 4 ℃. Then rinse with PBS for 5 min, 3 times. Add secondary antibody and incubate at 37 ℃ for 60 min. Finally, a microscope was used to observe and take pictures.

The linear density of VGluT I and VGAT immune response sites was analyzed using the ImageJ software (v1.34, National Institutes of Health, Bethesda, MD, USA) and expressed as the number of pincta/µm^2^ [29].

### Animals

Six male mice with autism disorder (*NL3*^R617W^ homozygous mutation) and six male SPF C57BL/6 mice (5-month-old, each 30 ± 5 g) were purchased from Zhejiang Weitong Lihua Co., Ltd. with SCXK (Jiaxing, China) 2021-0006 (Certificate No.: 20220824Abzz0600007602). The mice with autism disorder were assigned to the ASD group, while the SPF C57BL/6 mice were assigned to the control group. The mice were acclimatized in the animal house for 1 week before the experiment. Moreover, the mice were given water and food *ad libitum*, and kept in an environment with an ambient temperature of 20–25 ℃ and a relative humidity of 40%–70%. The experiments were carried out in accordance with the protocols approved by the Institutional Animal Care and Use Committee of Hangzhou Hibio Tech Co., Ltd. (HB210102). The mice were anesthetized by intraperitoneal injection of pentobarbital sodium (1.5%, 0.2 mL/100 g) and euthanized by inhalation of carbon dioxide.

### Behavioral Detection

All the animals were fed adaptively for 1 week, during which they were given normal access to food and water. The general conditions of the mice, including diet, hair, activity, and mental state, were observed and recorded. After the adaptive feeding, the Morris water maze (ZH006, Huaibei Zhenghua Biological Instrument Equipment Co., Ltd., Huaibei, China) and the Y maze (SA204, Jiangsu Sans Biotechnology Co., Ltd., Nanjing, China) experiment were conducted. The I and II arms of the Y maze were open, and the III arms were closed, and the mice were free to explore for 5 min. The III arm was opened after the above steps, and the free exploration was the same for 5 min. After all the mice had explored the maze, the experiment was repeated at an interval of 2 h.

The locomotor activity was assessed using the open-field test. The number of line crossings in a square maze was used to evaluate locomotor activity, and the act of defecating was thought to be a straightforward indicator of anxiety. The maze’s overall dimensions were 520 × 520 × 310 mm, with black Plexiglas sidewalls and a white Plexiglas floor divided into nine squares. The experiments were conducted in dim red light. The entire count of both vertical and horizontal line crossings was recorded throughout the first 5 min of the open-field test. When the head and limbs of the mouse cross the line, the mouse is considered to have crossed the line.

The three equal-sized chambers, each measuring 20 × 43 cm, were constructed in a rectangular Plexiglas box with two transparent partitions (Stoelting Co., Wood Dale, IL, USA). Entry to the chambers was facilitated via a central square aperture (8 × 5 cm) in each partition. The left and right rooms had cylindrical wire cages (7 cm in diameter) that either housed an inanimate object or a stranger mouse. The three-chamber test comprised two sections. The first section focused on evaluating social connection and sociability of the animals, while the second section assessed their social novelty and memory.

Two unrelated mice (stranger 1 and stranger 2) that matched the subjectmouse (control or ASD group)’s age and sex were needed for the test. A stranger mouse was excluded if exhibiting aggressive behavior. In every experiment, the same two stranger mice, which had never encountered the mouse under investigation, were utilized. All mice were relocated to the experimental room 30 min prior to the start of the studies. The subject mouse was first placed in the middle room and given 5 min to explore all the three compartments (habituation). Subsequently, a new mouse was introduced into one of the wire cages, and the subject mouse was given 10 min to investigate (session 1). The test mouse stayed in the test arena throughout the transition from session 1 to session 2.

In the session 2, the subject mouse was given an additional 10 min to interact with both stranger mice after another one was added to the previously empty wire cage. No temporal difference was observed between sessions 1 and 2 in our version of the model, except for the switching time. Session 2 began when stranger 2 mouse was positioned beneath the wire cup that had been left empty throughout session 1. After each trial, 85% ethanol was used to clean the wire cages and three-chamber equipment. Every experiment was carried out in low red light and captured using a camera.

The following behavioral metrics were examined by an observer, who was unaware of the mouse genotype:

(1) Number of entries in each chamber, indicating the locomotor activity. An entry was considered when the mouse’s head and four paws were inside the chamber. (2) Chamber time, indicating the amount of time spent in each chamber (with the head and all four paws). (3) Sniffing time, indicating the amount of time spent in the approximately 2 cm proximal zone of the wire cage with the head oriented towards it. Perching on the wire cage was not regarded as appropriate social conduct.

These behaviors were analyzed with EthoVision software v1.90 (Noldus, Wageningen, Netherlands).

### Statistical Analysis

Data analysis was performed using GraphPad version 9.0 statistical software (GraphPad Software, Inc., New York City, NY, USA). Multiple comparisons between multiple sets of data are conducted using one-way analysis of variance (ANOVA) with Tukey’s test for post-hoc analysis. For comparison between two sets of data, the *t*-test was utilized. Descriptive statistics are presented as mean ± standard deviation for continuous variables. *p* < 0.05 indicates that the result was statistically significant, *p* < 0.01 indicates high statistical significance, and *p* < 0.001 indicates very high statistical significance.

## Results

### Membrane and Intracellular Distribution of NL3 in the Cells Transfected with NL3-HA-WT or NL3-HA-R617W

**Fig. 1.**
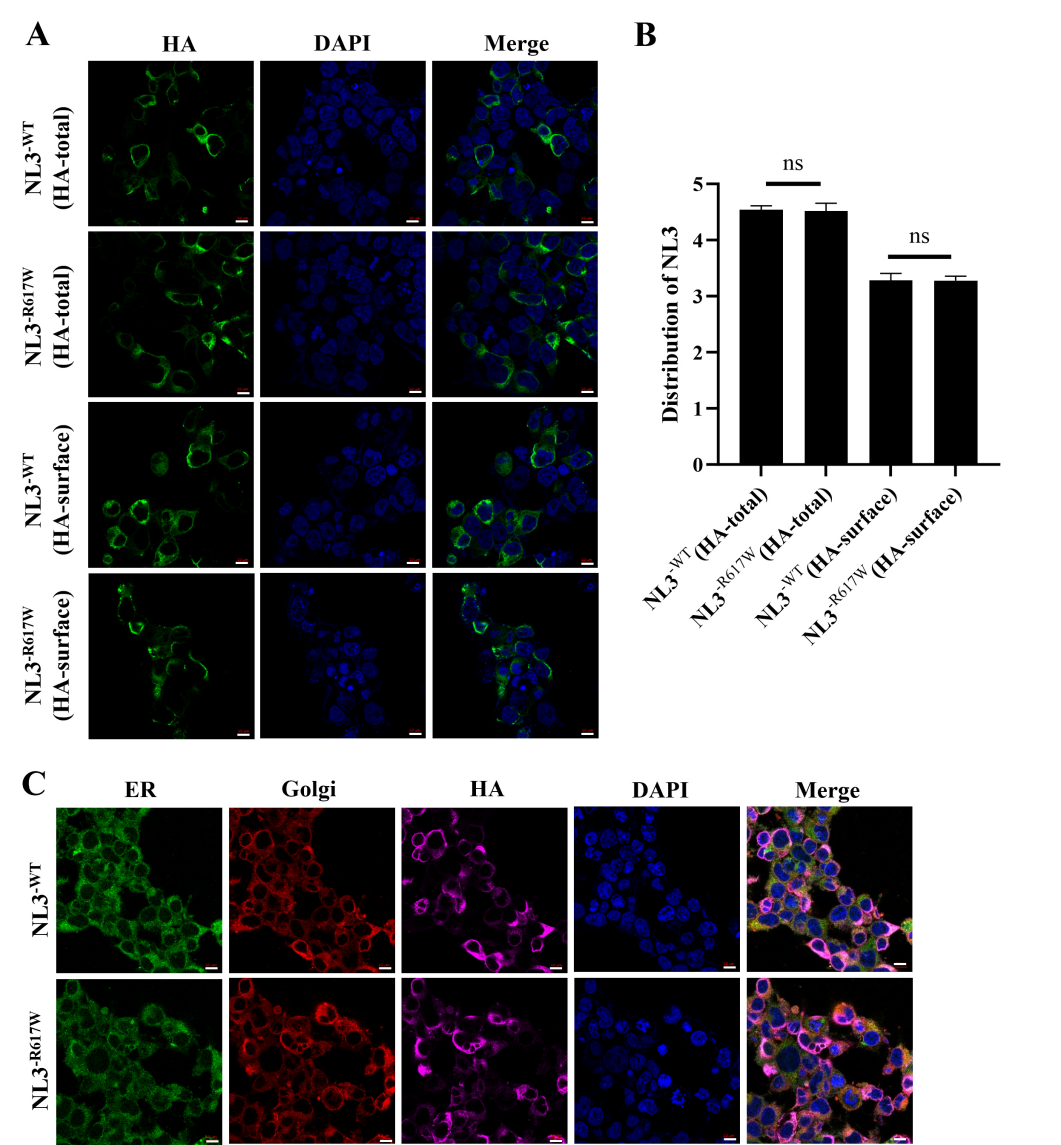
**Distribution of NL3**. (A,B) Expression of NL3 in the cell membrane. (C) Confocal laser microscopic images showing NL3 distribution in the cell and endoplasmic reticulum. Scale bar: 10 µm. Magnification: 200×. NL3, neuroligin-3; HA, hemagglutinin; HA-total, the total expression of HA in cells; HA-surface, the expression of HA on the cell membrane; ER, endoplasmic reticulum; Golgi, Golgi-tracker; DAPI, 4’,6-diamidino-2-phenylindole. n = 6. ns, no significant difference.

Fig. 1A,B illustrates no discernible variation in the membrane NL3 expression in the 293T cells transfected with NL3-HA-WT or NL3-HA-R617W, regardless of membrane integrity (*p* > 0.05). Specifically, the *NL3*^R617W^ mutation did not influence the expression of NL3 membrane. The 293T cells transfected with NL3-HA-WT or NL3-HA-R617W did not exhibit any appreciable variations in the intracellular and endoplasmic reticulum distribution of NL3 (Fig. 1C). Hence, the *NL3*^R617W^ mutation had no effect on the intracellular distribution of NL3 and did not result in endoplasmic reticulum retention.

### *NL3*^R617W^ Mutation Affects the Interaction of NL3 with NRXN1 in the 293T Cells

**Fig. 2.**
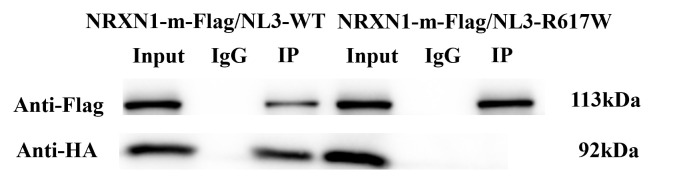
**The *NL3*^R617W^ mutation affects the interaction of NL3 with NRXN1**. n = 6. NL3, neurexin 1; IgG, immunoglobulin G; IP, immunol precipitation.

Further anti-HA immunoblotting (IB) detection of input, Ig, and IP products indicated that a positive signal corresponding to a 92 kDa protein (HA) was detected in NRXN1-m-FLAG/NL3-WT sample (Fig. 2). Meanwhile, the expression of this protein was not detected in NRXN1-m-FLAG/NL3-R617W sample. This notion suggests that NL3 interacts with NRXN1, and *NL3*^R617W^ mutation has an impact on the interaction between NL3 and NRXN1.

### *NL3*^R617W^ Mutation Reduces Synaptic Excitability

**Fig. 3.**
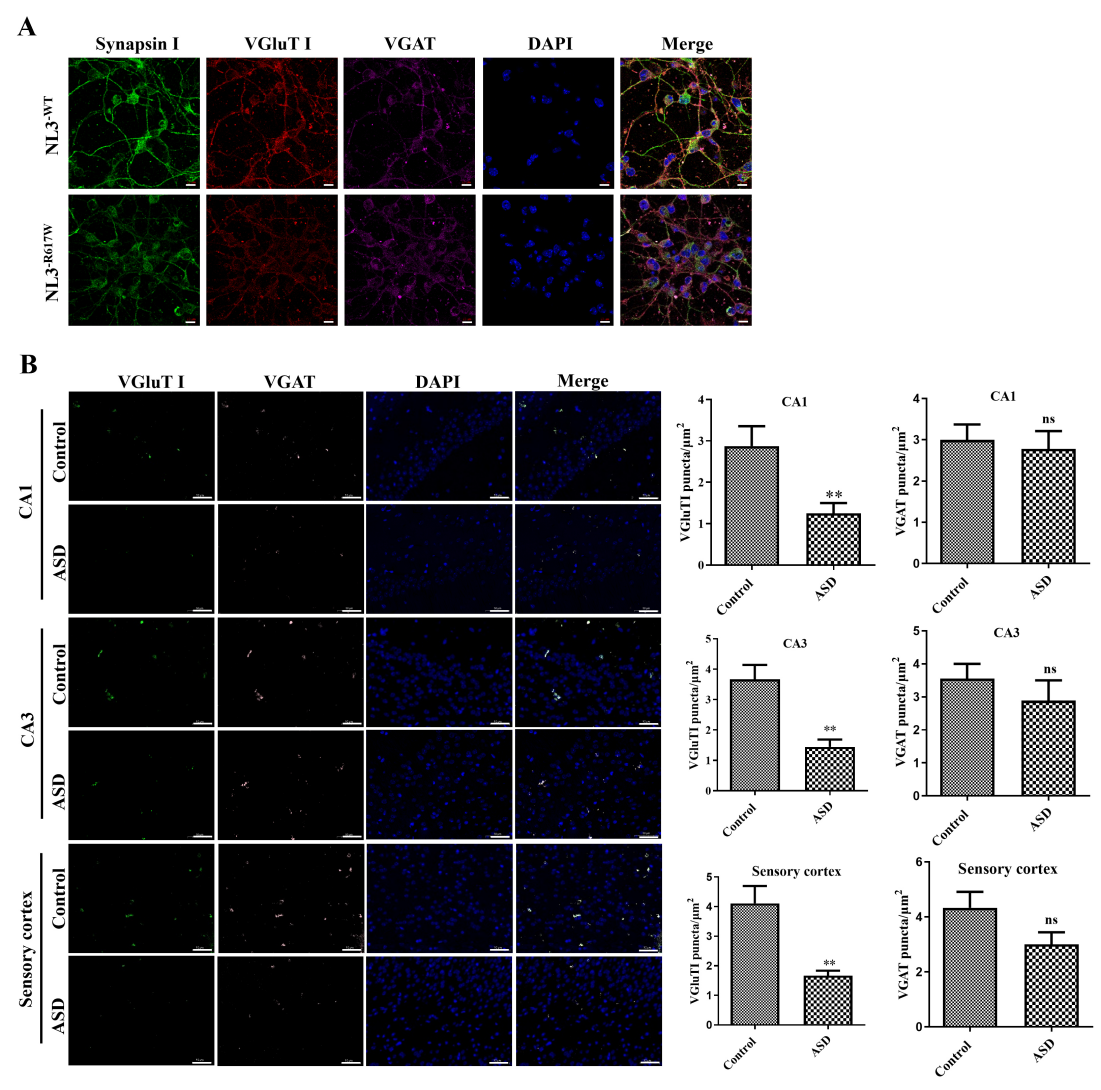
**The *NL3*^R617W^ mutation reduces synaptic excitability in neurons**. (A) Confocal microscopic images showing expression of Synapsin I, vesicular glutamate transporter (VGluT) I and vesicular γ-aminobutyric acid transporter (VGAT) in neurons. Scale bar: 10 µm. (B) Confocal fluorescence microscopic images showing the expression of cornu ammonis (CA)1 and CA3 in hippocampus and VGluT I and VGAT in sensory cortex of autistic mice, and the corresponding quantitative analysis. Scale bar: 50 µm. Magnification: 200×. n = 6. ns, no significant difference; **p < 0.01.

Synapsin I is regarded as a prominent plasticity marker in neural networks. VGluT I is a neuron-expressed protein involved in the vesicle circulation of neurotransmitters. VGAT is a common vesicular transporter of γ-aminobutyric acid (GABA) and glycine and is essential for normal GABA and glycinergic neurotransmission. The Synapsin I and VGluT I in the *NL3*^R617W^ group decreased in expression when compared with the *NL3*^𝑊𝑇^ group, while there was no significant difference in VGAT detected between the two groups (Fig. 3A). Compared with the control group, the positive expression of VGluT I of cornu ammonis (CA)1, CA3 and sensory cortex in the brain of mice of the ASD group was significantly decreased (*p* < 0.01), and the positive expression of VGAT was not significantly different (*p* > 0.05) (Fig. 3B).

### Water Maze and Y Maze Experiments

**Fig. 4.**
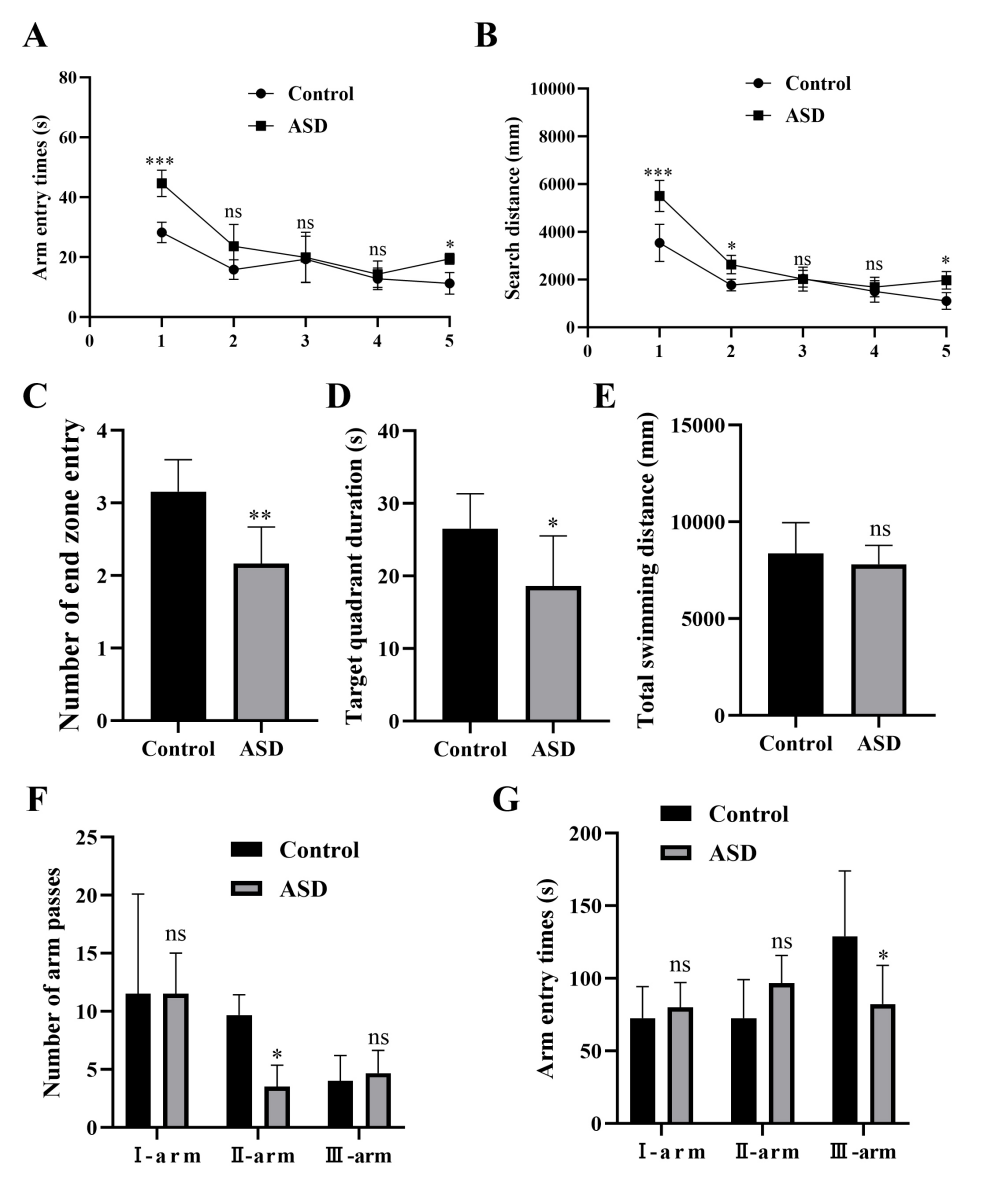
**Behavioral testing**. (A–E) The comparative analysis of parameters measured in the water maze experiment: detection latency (A), search distance (B), number of end zone entry (C), target quadrant duration (D), and total swimming distance (E). (F,G) The comparative analysis of parameters measured in the Y maze experiment: number of passes at the I, II and III arms (F), and entry times through I, II and III arms (G). ASD, autism spectrum disorder. n = 6. ns, no significant difference; *p < 0.05; **p < 0.01, ***p < 0.001.

As shown in Fig. 4A,B, on the first day of the plateau period, the incubation period and search distance of mice in the ASD group were significantly higher than those in the control group (*p* < 0.001). The search distance of mice in ASD group was significantly higher than that in control group on the second day (*p* < 0.05), and the latency and search distance of mice in ASD group were significantly higher than that in control group on the fifth day (*p* < 0.05). As shown in Fig. 4C–E, after the withdrawal of the platform, the mice of the control group had significantly longer swimming time in the target quadrant and more entries into the end zone than the ASD group (*p* < 0.01 and *p* < 0.05, respectively), and the total distance was found to be slightly longer in the control group than that in the ASD group, despite no statistical significance (*p* > 0.05). In Fig. 4F,G, the Y maze experiment results showed that the number of Ⅱ-arm entry times in the ASD group was significantly lower than that in the control group (*p* < 0.05), and the Ⅲ-arm entry time was significantly reduced in the ASD group compared to the control group (*p* < 0.05).

### Open-Field Test

**Fig. 5.**
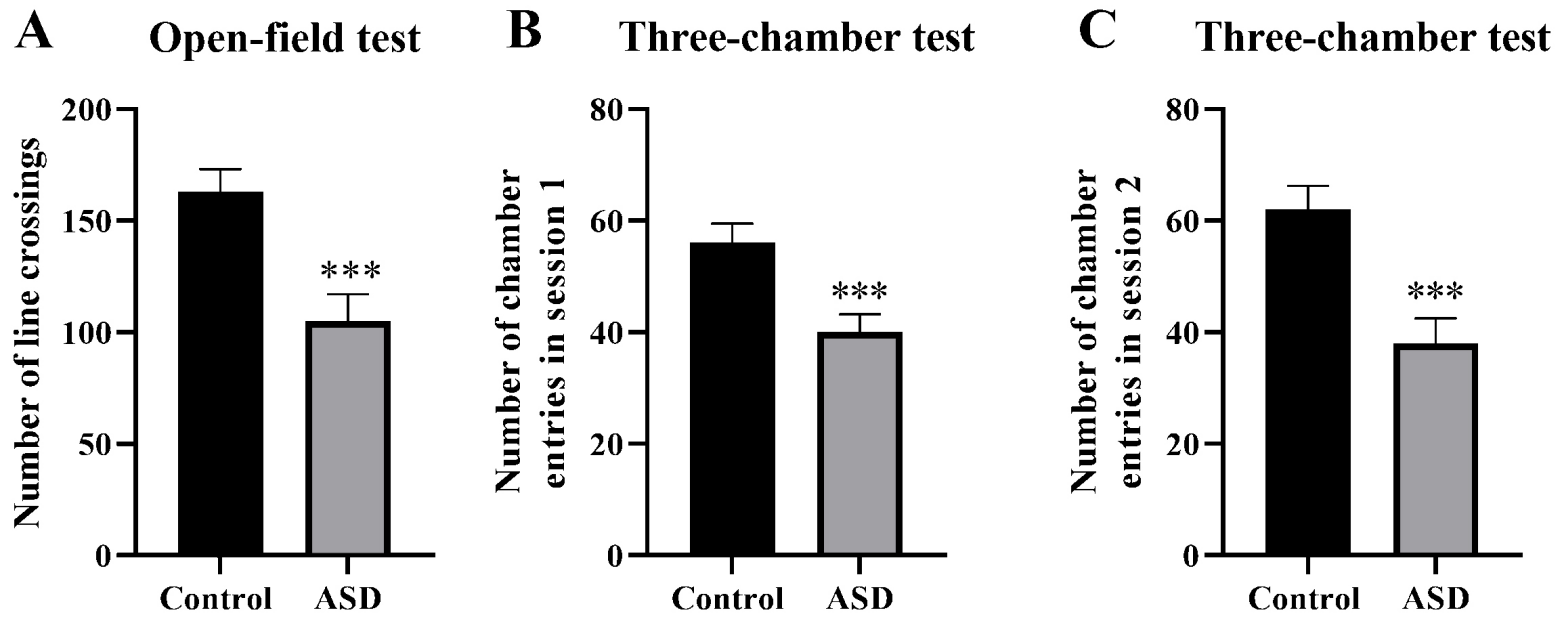
**Locomotor activity measured in the open-field (A) and three-chamber tests (B,C)**. n = 6. ***p < 0.001.

According to the open-field test results, the ASD mice became less active than the control mice, following a half-hour exercise, as revealed by a significantly lower number of line crossings (*p* < 0.001, Fig. 5A–C).

### Three-Chamber Social Test

**Fig. 6.**
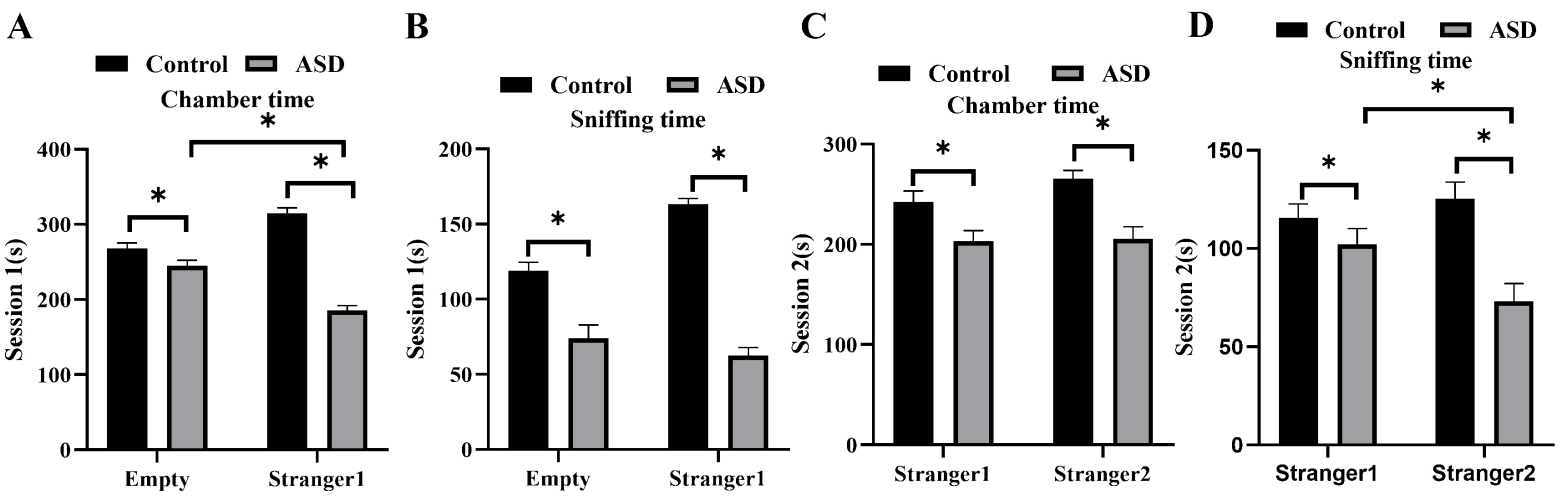
**Three-chamber social test results on social interaction and social novelty**. (A,B) Results from session 1: average chamber times for social affiliation and sociability (A), and sniffing duration (B). (C,D) Results from session 2: average chamber times for social affiliation and sociability (C), and sniffing duration (D). n = 6. *p < 0.05.

The chamber time (amount of time spent in the wire cage-containing chambers) and sniffing time (amount of time spent sniffing the wire cages) were measured to evaluate the mice’s social behavior. The test was divided into two sessions: session 1 measured friendliness, while session 2 assessed memory and social novelty. In session 1, the ASD mice spent shorter time in the empty cage than the control mice (*p* < 0.05), and significantly lesser time in the cage housing the stranger mouse 1 (*p* < 0.05, Fig. 6A). In Fig. 6B, the sniffing time of ASD mice in the cage housing stranger mouse 1 was significantly shorter than that of control mice, and the same trend was also observed in the empty cages (*p* < 0.05). In session 2, the ASD mice also spent significantly shorter time staying and sniffing in the chamber housing stranger mouse 2 (*p* < 0.05, Fig. 6C,D). In addition, the ASD mice spent significantly lesser sniffing time in the chamber housing stranger mouse 2 than the one housing stranger mouse 1 (*p* < 0.05). These results may suggest that the ASD mice did not have a preference for unfamiliar mice, in terms of social interaction.

### Synaptic Protein Reduction in the NL3^R617W^ Mutant Mice

**Fig. 7.**
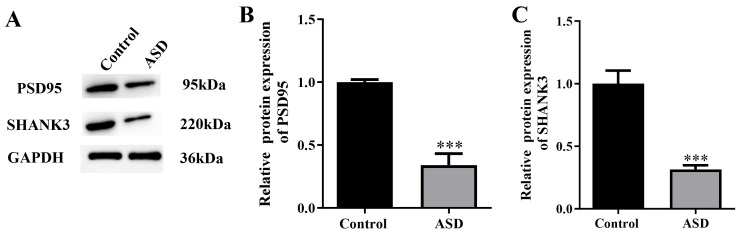
**Synaptic protein reduction in *NL3*^R617W^ mutant mice**. (A) Expression of postsynaptic density protein-95 (PSD95) and Src homology domain and multiple ankyrin repeat domains protein 3 (SHANK3) proteins detected by means of Western blotting. (B,C) Protein expression quantitation of PSD95 (B) and SHANK3 (C). GAPDH, glyceraldehyde-3-phosphate dehydrogenase. n = 6. ***p < 0.001.

The protein expression of PSD95 was significantly decreased in the ASD group than in the control group (*p* < 0.001, Fig. 7A–C). Similarly, the SHANK3 protein expression was also significantly decreased in the ASD group, compared with that in the control group (*p* < 0.001).

## Discussion

In this study, we discovered for the first time the impact of *NL3*^R617W^mutation on autistic mice. We found that the *NL3*^R617W^ mutation influences the interactions of NL3 with NRXN1, instead of membrane expression or endoplasmic reticulum retention of NL3. Furthermore, the number of synapses and the expression of postsynaptic scaffold proteins, PSD95 and SHANK3, were found to be decreased in autism associated with *NL3*^R617W^ mutation.

Retention of working memory relies on the prefrontal cortex and the hippocampus to store and manipulate transient information [30, 31]. The Y maze is a spatial memory test that takes advantage of rodents’ innate propensity to alternate spontaneously in each of the maze’s three arms with a success rate of approximately 60%–70% [32]. Additionally, the Morris water maze, a well-known test to evaluate spatial learning and memory of rats [33, 34], was used in this investigation. Our findings showed that memory and learning ability were decreased in the *NL3*^R617W^ mutation group. Our results highlight that motor activity might be a useful measure for evaluating habituation in the open field, which is frequently investigated in rodent studies [35, 36]. A previous study reported that the *Shank3b* homozygous knockout mice exhibited impaired sociability and excessive/injurious self-grooming in the three-chamber test [37].

Our findings suggested that mice in the* NL3*^R617W^group exhibited a preference for social contact but concurrently showed a decrease in social ability. This, however, presents contradiction to the prevailing perception that *NL3*^R617W^ is autism-associated, and thus, careful analysis is required to identify potential explanations. Firstly, the preference for social contact observed in mice of the *NL3*^R617W^ group may indicate an enhanced social inclination to some extent. This could be related to the impact of the mutation on neural system function, possibly enhancing certain types of social behaviors or eliciting stronger responses to social stimuli. However, the presence of social preference does not necessarily imply an enhancement in social ability, as it typically refers to appropriate behaviors and capabilities in social interactions. Secondly, our research findings showed a decrease in social ability among the mice in the *NL3*^R617W^ group, which is unveiled by the poorer performance, in terms of lower frequency or quality of interaction with peers, in standard social behavior tests. The lower social ability manifested by the mutant mice may be associated with the neurobiological changes induced by the mutation, which could affect social communication and interaction among the mice. The findings from this study highlight the complexity in the effects of *NL3*^R617W^ mutation on social behavior of the mutant mice. Despite a preference for social contact, these mutant mice exhibited a decline in social ability. Taken together, further in-depth studies on the effects of this mutation on social behavior and its underlying neurobiological mechanisms are warranted.

NL3 plays a role in the excitatory and inhibitory synaptic transmission inside the brain [38]. However, there has been a limited knowledge concerning the distribution of NL3 protein in organisms harboring the *NL3*^R617W^ mutation. Our study revealed that the *NL3*^R617W^ mutation did not affect the distribution of NL3.

In addition to the functions as scaffolding proteins at excitatory synapses, PSD proteins like PSD-93 and PSD-95 recruit α-amino-3-hydroxy-5-methyl-4-isoxazole-propionicaci (AMPA) receptors and bind N-methyl-D-aspartic acid receptor (NMDA) receptors, all of which affect neurotransmission [39]. PSD-95 also binds to neuroligins to facilitate the recruitment of ion channels and receptors to the synapse [40, 41]. Accordingly, SHANK3 is essential for a number of neuroplasticity features during development [42]. Interactions between the “SAPAP” (synapse-associated protein (SAP) 90/PSD-95-associated protein) family and the postsynaptic density 95 (PDZ) SHANK domain [43]. The role of PSD-95 and the interaction profile of SHANK3 were validated in this work by examining their protein levels in the striatal brain tissues, including those from hippocampus, thalamus, amygdala, and corpus callosum [44]. Contrary to the predicted sharp decline in NL3 in the striatal brain tissues examined, no significant changes in PSD-95 abundance were observed in the cortex or cerebellum, indicating that enhanced PSD-95 protein expression is unique to the striatum [45]. In the current study, we also detected significant reductions in the expression levels of postsynaptic scaffolding proteins SHANK3 and PSD95, which are involved in promoting the release of new transmitters, in mice harboring the *NL3*^R617W^ mutation.

Based on the available data, *NL3*^R617W^ mutation is possibly involved in the formation of synapses and the modification of synaptic function during neuroglia contact in the hippocampal regions of mice. An altered excitatory/inhibitory balance was observed in the hippocampus CA1 pyramidal neurons in mice with SHANK1 defect, which is regarded as a pathophysiologic characteristic of ASD. In addition, the presence of* NL3*^R617W^ mutation lowers CA1 and CA3 neuron activity in mice, consistent with findings obtained in various ASD models [46, 47, 48].

The hippocampal CA1 and CA3 and the sensory cortex of the *NL3*^R617W^group suffered a tremendous decline in the number of excitatory synapses, with the inhibitory synapses spared from the decimation. This finding is noteworthy because it indicates a correlation between *NL3*^R617W^ mutation and autism-like behavior in autistic animals. On this basis, we suggest that the behavioral characteristics under investigation are probably triggered by the *NL3*^R617W^ mutation that leads to reduced spontaneous synapse transmission.

Certain abnormalities related to spatial memory and exploration abilities were observed in the *NL3*^R617W^mutant mice, compared to the control mice. Latency, search distance, total distance traveled, approach times, and time are some of these disruptions. The behaviors that lengthen the search delay observed in these animals are assumed to have been caused by the *NL3*^R617W^ mutation-induced motor inhibition. A reduction in the maze scores is an indication of worsening sensorimotor abnormalities [49]. These results underscore the importance to thoroughly screening for *NL3*^R617W^ mutation in the treatment of any sensory and memory deficits manifested in autistic mice.

## Conclusions

In summary, this work successfully established a mouse model of autism associated with the *NL3*^R617W^ mutation, which triggers cognitive and memory impairments resembling the characteristics observed in individuals with autism. These findings underline the therapeutic value of targeting the *NL3*^R617W^ mutation and shed light on the pathophysiology of autism related to *NL3* mutations.

## Availability of Data and Materials

The original contributions presented in the study are included in the article. Further inquiries can be directed to the corresponding author.
